# Supplementary material for: Non-equilibrium dynamics of a nascent polypeptide during translation suppress its misfolding
Source: Nat Commun. 2019 Jun 20;10:2709. doi: 10.1038/s41467-019-10647-6 (PMC6586675; doi:10.1038/s41467-019-10647-6)
Supplement: Supplementary file 1 — Supplementary Information [file 41467_2019_10647_MOESM1_ESM.docx]

**Supplementary Information**

**Non-equilibrium Dynamics of a Nascent Polypeptide During Translation Suppress its Misfolding**

**Alexander et al.**


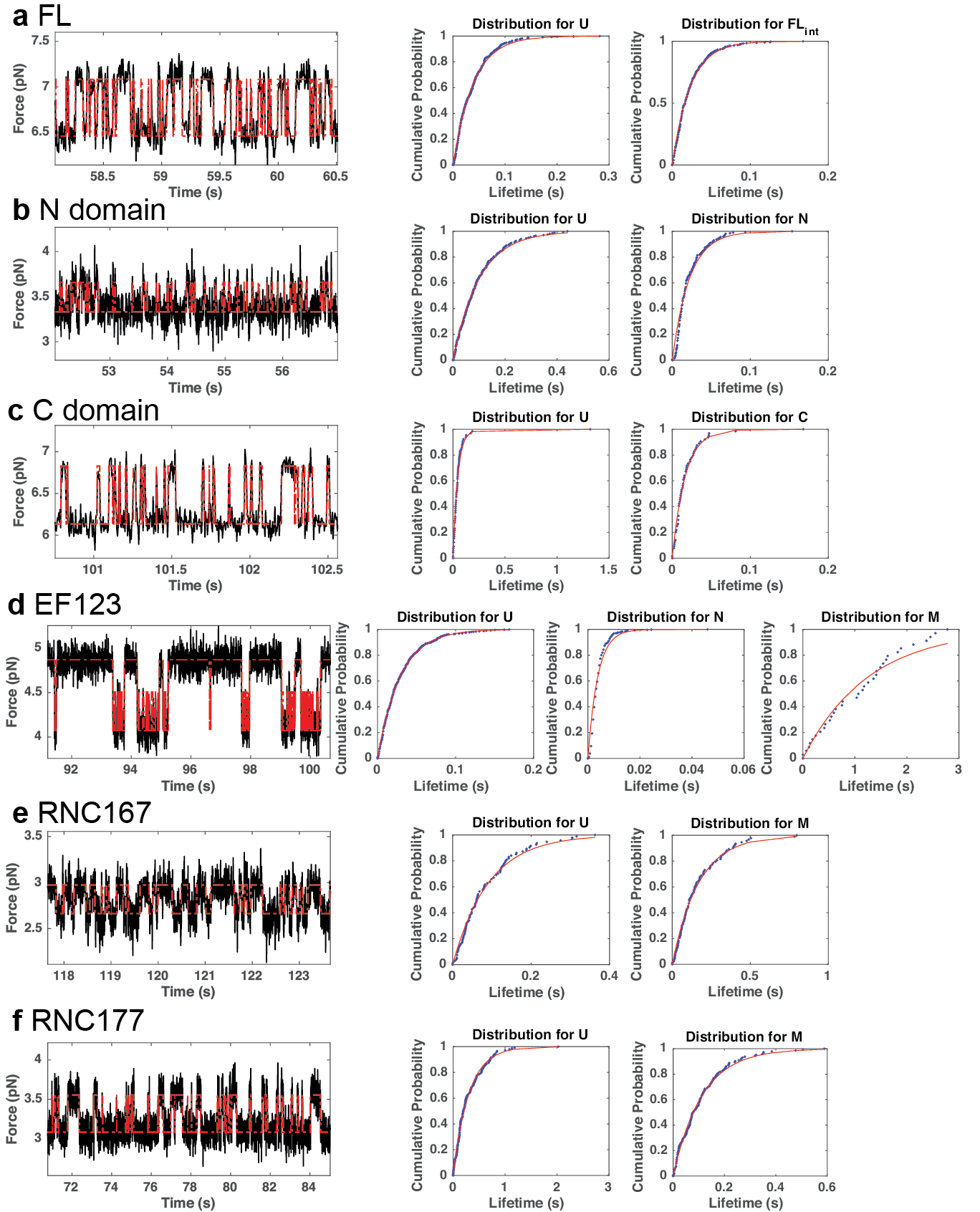


Supplementary Figure 1: Example CDFs from the HMM fit for each construct. If the HMM adequately describes the underlying kinetic system, all of the fits should be exponential. Example passive molecules are shown with a HMM overlaid (red dashed lines). The CDFs are fit with exponentials (red lines). **a** the FL protein. **b** N domain. **c** C domain **d** EF123 construct **e** RNC167 **f** RNC177. Source data are provided as a Source Data file.

**
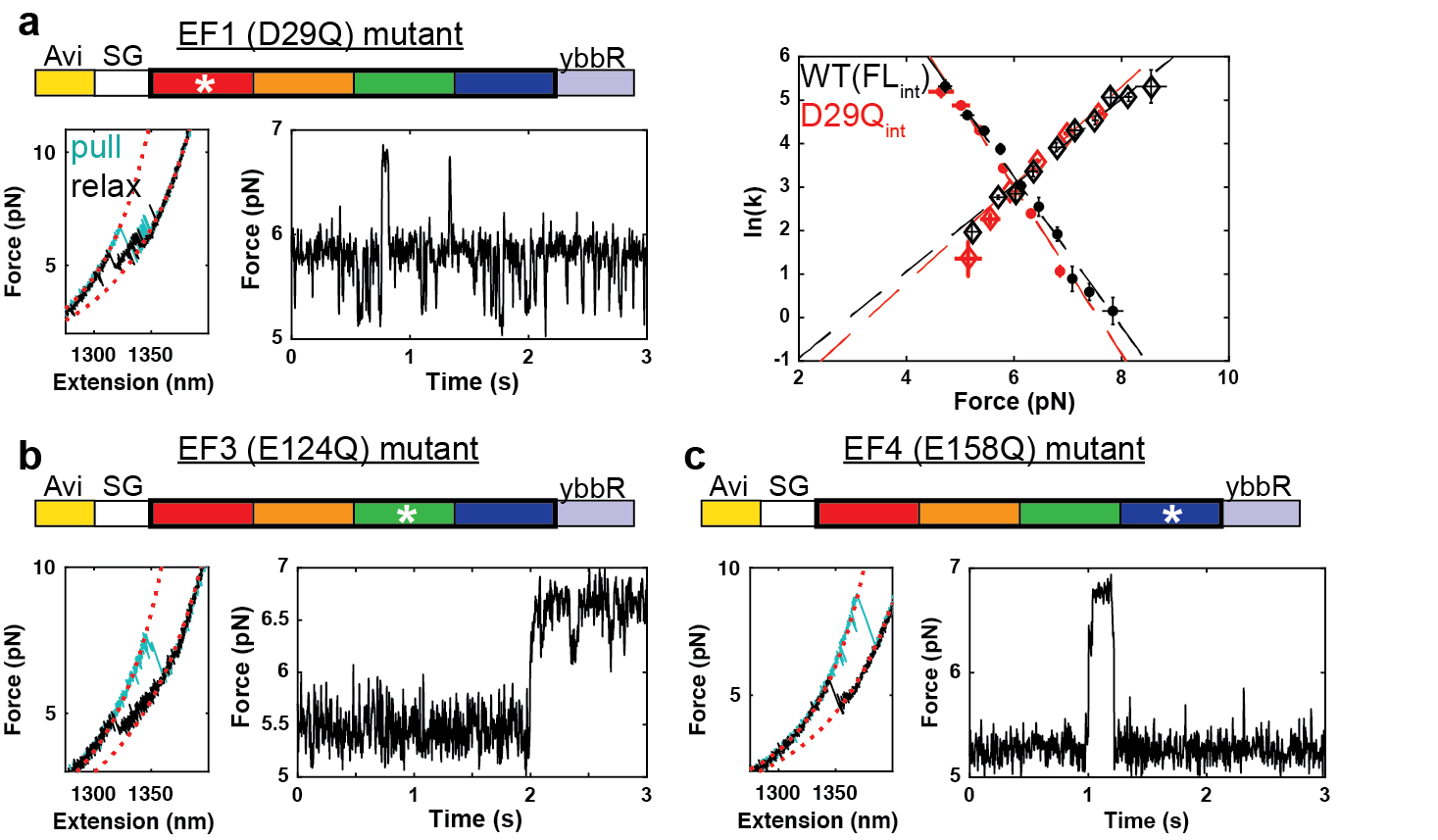
**

Supplementary Figure 2: Calcium-binding mutants also confirm C-domain intermediate. **a** The EF1 mutant (D29Q) shows two-step (non-cooperative) unfolding and faster unfolding from folded state to the intermediate, but no effect on the intermediate’s kinetics (compare FL_int_ to D29Q_int_), consistent with a C domain intermediate. Error bars are SE. For D29Q, n =7 molecules, 63 rate measurements. Cyan is the unfolding curve, black is refolding. Red dotted lines are WLC of DNA and WLC of DNA+177 amino acids of polypeptide. **b** The EF3 mutant (E124Q) destabilizes the intermediate (folding begins at ~5 pN instead of 7 pN). In addition, the fully folded state shows new partial unfolding transitions in passive mode, which were not observed for the WT (the downwards jumps from the state at 6.8 pN). Representative from n = 11 molecules. **c** The EF4 mutant (E158Q) shows the same qualitative behavior as the EF3 mutant, but refolding occurs at even lower forces. A similar partial unfolding of the fully-folded state is seen. Representative from n = 11 molecules. Apo-calerythrin has been observed to be a molten globule^21^ and it is likely that the fully folded state of E158Q and E124Q are not native conformations, hence why the EF hands are not stably bound. Source data are provided as a Source Data file.


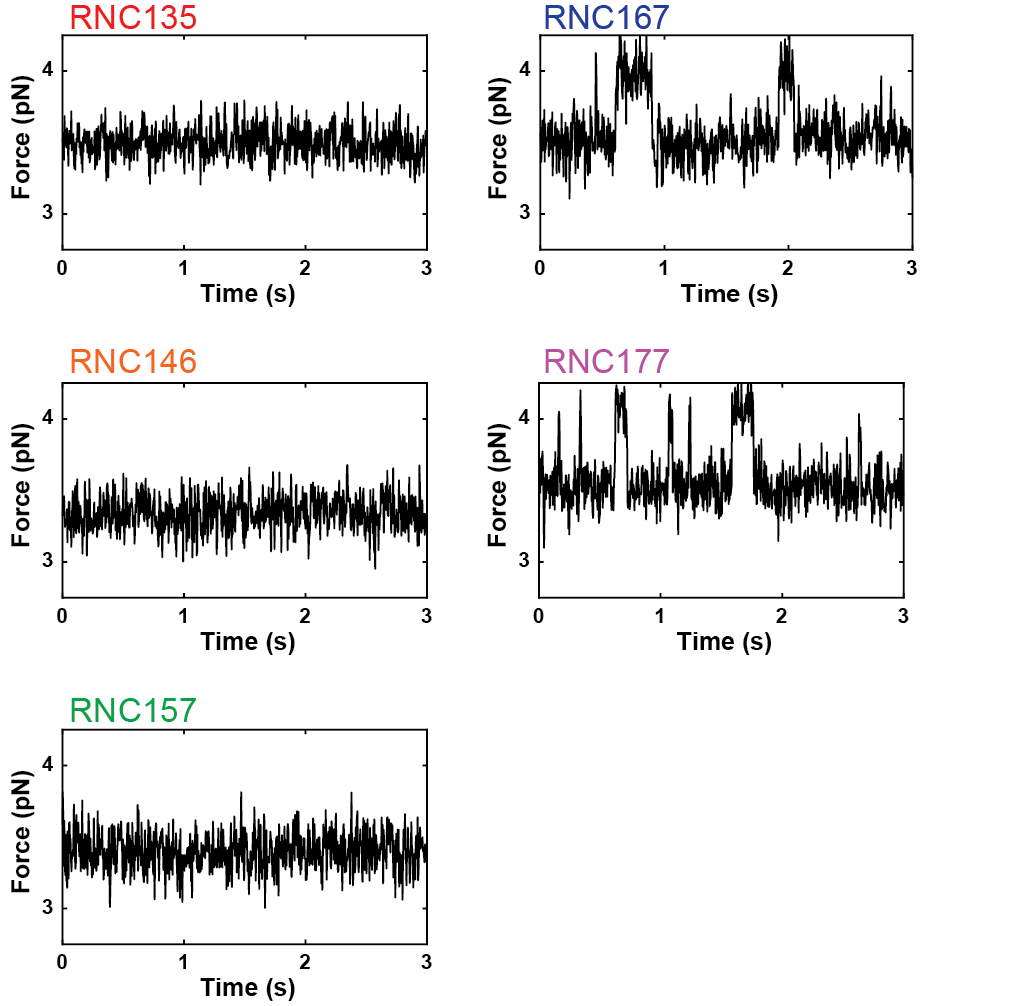


Supplementary Figure 3: Passive measurements on the RNCs. Although force extension curves are a more comprehensive way to demonstrate a lack of folding, since they cover the full range of forces and the pulling bias tends to increase the force at which unfolding occurs, we can also probe a range of forces with passive data collection. In these examples where the unfolded state is near 3.5 pN, we see no folding for RNCs 135, 146, and 157. From 3.5 pN for the unfolded state, the N-domain transition is expected to be to 3.8 pN and the misfolded state would be at 4.0 pN. RNC167 and RNC177 fold to the misfolded state. Representative from n = 3, 10, 9, 5, 10 molecules respectively. Source data are provided as a Source Data file.


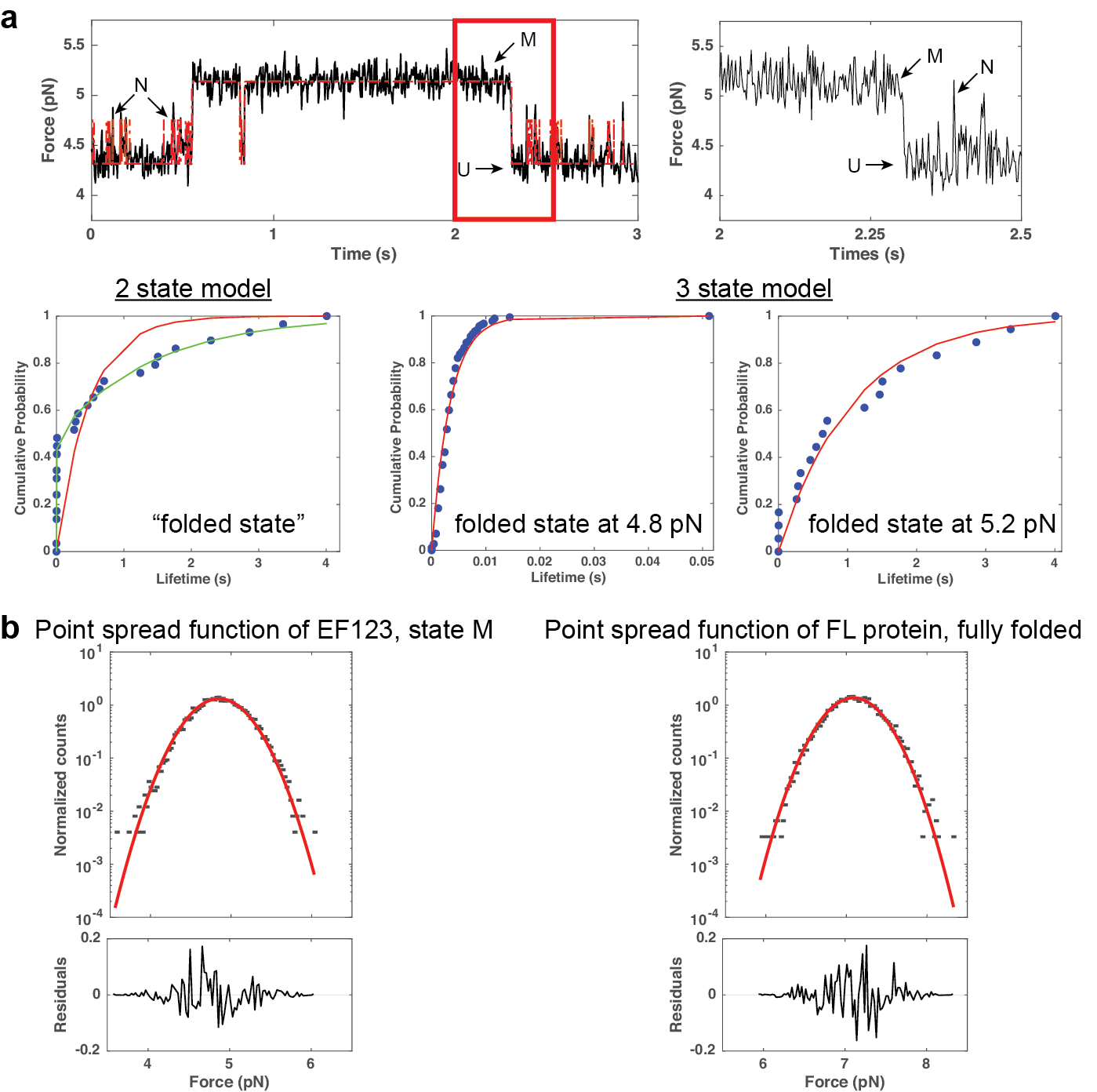


Supplementary Figure 4: EF123 is a 3-state system with an off-pathway state. **a** The passive mode data shows a long lived misfolded state (M) and a short-lived intermediate-length state (N domain) from the unfolded state (U). Red dashed line is the HMM fit. A zoom-in of the passive data shown at higher bandwidth (267 Hz) shows the short-lived intermediate state. Fitting a 2-state HMM results in skewed double exponential distributions for the folded state (green line), which is an incorrect combination of N and M transitions. A 3-state model gives exponential distributions for both N and M (red lines). **b** The point-spread function of state M does not show any skew at lower forces, which would be indicative of transitions to state N, thus corroborating the HMM assignment that N and M are not connected. The point-spread function of the FL protein does not show any skew at lower forces either, indicating that M is not accessible from the FL protein and it is indeed off-pathway.


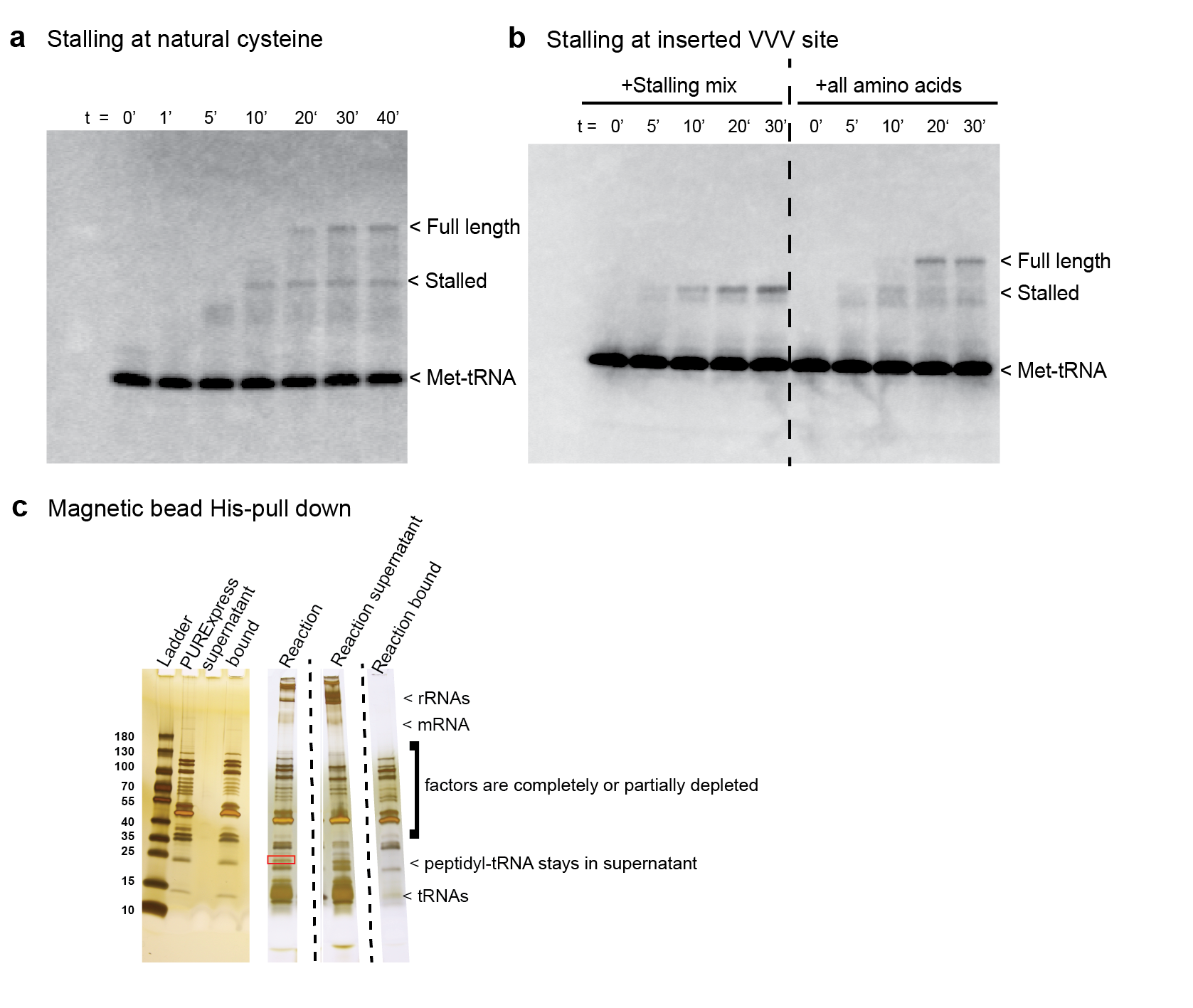


Supplementary Figure 5: Generating stalled complexes for real-time. **a** Stalling via amino acid omission is prone to read-through of the stall site, as measured through ^35^S-Met translation timecourses. We tested stalling via a single omitted amino acid (the natural cysteine in calerythrin) before designing the new mRNA. The stall RNC accumulates after 10’, however, by 20’ a mixture of stall and read-through is present. Based on this, we decided to design a stall site of 3 sequential codons to ensure long-lived stalling. **b** The complete sequence we use in our experiments, which contains 3 sequential valines, does not show read-through up to 30 minutes in the presence of the stalling mix components. Running an experiment with all 20 amino acids on the same gel shows that the read-through band is distinguishable. The faint band underneath the stalled band and in the all amino acid condition may be due to slower translation through the serine-glycine linker. **c** After stalling, the protein components of the stalling mix are removed via a His-pull down. As shown, the beads pull down all components of the PURExpress kit when ribosomes are not present (bound sample). When ribosomes are included (reaction condition), the beads pull down all of some bands and partially deplete others. The ribosome, tRNAs, and nascent chain are not present in the bound sample and remain in the supernatant as expected. Although some factors may come through the pull down, it is not enough for multi-turnover translation and the complexes can be kept on ice for experimentation. Source data are provided as a Source Data file.

Supplementary Figure 6: Finding first folding transition in real-time data. The real time-data was divided into 1 second windows. Within each window, the data was downsampled by a factor 10, then each point was compared to the mean via a Gaussian CDF. Top panel shows the data downsampled by 10 to 133.33 Hz. The black line is the fit to translation, the green x marks codon 167. The red dots mark points flagged as transitions; we only care about the first transition. The middle panel shows the computed probability and cutoff (red dashed line). The third panel shows the standard deviation in the window, which spikes in the case of long or frequent transitions, and is also flagged.

Supplementary Figure 7: The non-equilibrium delay is best modeled as an exponential function. The experimental data is shown as blue circles. The single exponential fit is shown in red, the double exponential fit is shown in green, and the Erlang distribution in magenta. The other fits are not better, according to an f-test. For the double exponential, p = 0.9999. For the Erlang, p = 0.3242.


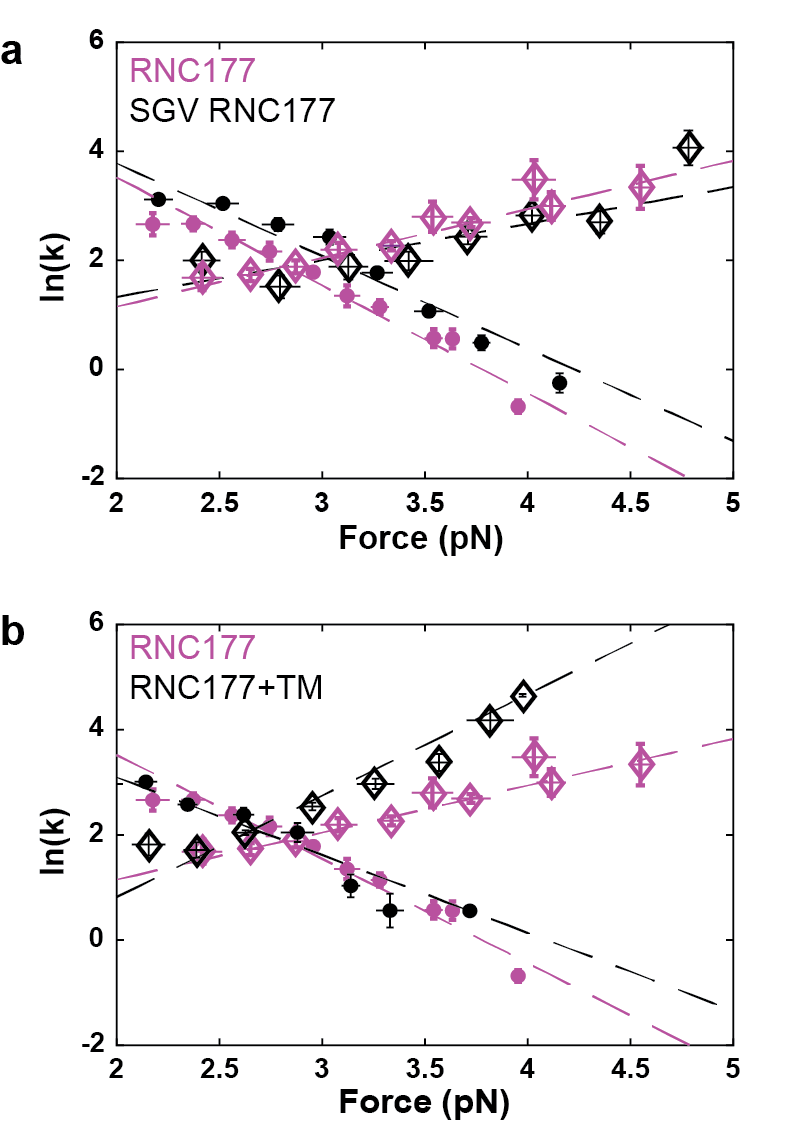


Supplementary Figure 8: The non-equilibrium delay is not due to the addition of the stalling sequence (labeled SGV for serine-glycine-valine) or translation mix (TM). **a** The addition of the sequence used for real-time stalling does not affect folding. RNCs stalled at codon 177 including the SGV linker (SGV RNC177) and without the linker (RNC177) show the same hopping behavior. The force-dependent kinetics do not change— *p* values for k_0, folding_ and k_0, unfolding_ show changes from RNC177 are not significant (>0.05; 0.35 and 0.24 respectively). Error bars are SE. RNC177: n = 10 molecules, 111 rate measurements; SGV RNC177: n = 7 molecules, 79 rate measurements. **b** The force-dependent kinetics of RNC177 show small changes in the presence of TM (see Supplementary Table 3) but not non-equilibrium behavior. Error bars are SE. RNC177: n = 10 molecules, 111 rate measurements; RNC177 + TM: n = 5 molecules, 68 rate measurements. Source data are provided as a Source Data file.


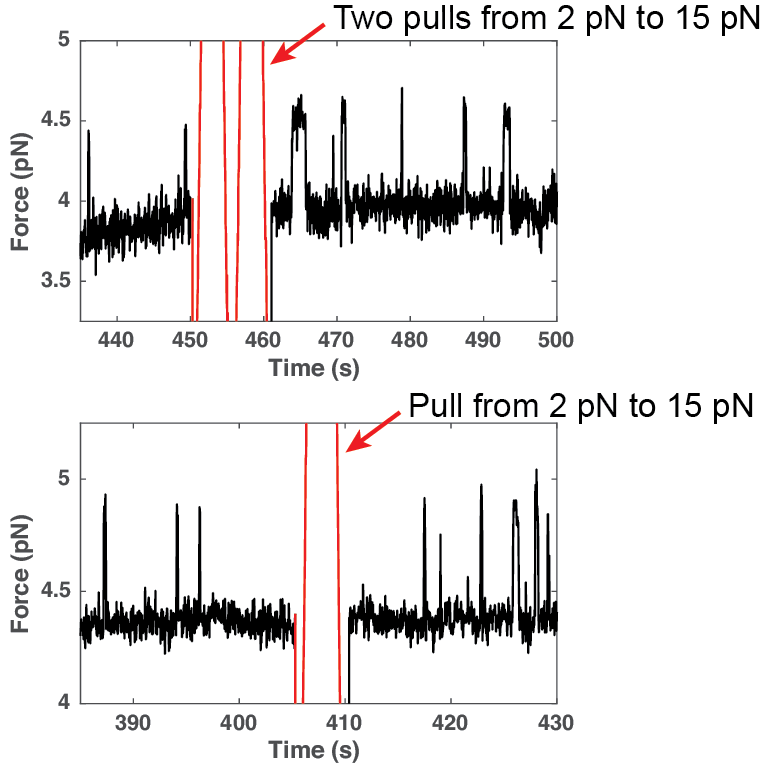


Supplementary Figure 9: The non-equilibrium delay is only observed upon initial synthesis. After molecules show hopping, raising the force to 15 pN or lowering the force to 2 pN (red data) does not induce the delay again, and hopping resumes. Source data are provided as a Source Data file.

Supplementary Table 1: Fit and extrapolated parameters of FL_int­_, C domain, and N domain. Showing 95% CI.

|  | Size  (amino acids) | k­_0, folding_  (s^-1^) * | *p* value compared to FL_int_ | τ_0, unfold_ (s) * | ∆x^†^_folding_ (nm) | *p* value compared to FL_int_ |
| --- | --- | --- | --- | --- | --- | --- |
| FL_int_ | 76 ± 6 | 7.7 x10^5^ (10^5.9±0.4^) | n/a | 1.3 x10^-6^ (10^-5.9±0.4^) | 7.1 ± 0.6 | n/a |
| C domain | 72 ± 8  Expected: 87 | 1.8 x10^6^ (10^6.3±0.5^) | n.s. (0.11) | 5.5 x10^-7^ (10^-6.3±0.5^) | 7.9 ± 0.8 | n.s. (0.06) |
| N domain | 98 ± 12  Expected: 90 | 660  (10^2.8±0.2^) | 2.6 x10^-10^ | 1.5 x10^-3^  (10^-2.8±0.2^) | 5.6 ­± 0.6 | 0.002 |
|  |  |  |  |  |  |  |
|  |  | k­_0, unfolding_ (s^-1^) * | *p* value compared to FL_int_ | τ_0, fold_ (s) * | ∆x^†^_unfolding_ (nm) | *p* value compared to FL_int_ |
| FL_int_ |  | 5.5 x10^-2^ (10^-1.3±0.2^) | n/a | 18  (10^1.3±0.2^) | 4.1 ± 0.3 | n/a |
| C domain |  | 3.1x10^-2^ (10^-1.5±0.3^) | n.s. (0.10) | 33  (10^1.5±0.3^) | 4.6 ± 0.4 | 0.04 |
| N domain |  | 1.2  (10^0.1±0.3^) | 2.7 x10^-6^ | 0.8  (10^-0.1±0.3^) | 3.9 ± 0.8 | n.s. (0.33) |

n.s.: not significant

Supplementary Table 2: Fit and extrapolated parameters of EF123 and RNC177. Showing 95% CI.

|  | Size  (amino acids) | k­_0, folding_  (s^-1^) | *p* value compared to RNC177 | τ_0, unfold_ (s) | ∆x^†^_folding_ (nm) | *p* value compared to RNC177 |
| --- | --- | --- | --- | --- | --- | --- |
| RNC177 | 130 ± 15 | 1.8 x10^3^ (10^3.3±0.2^) | n/a | 5.6 x10^-4^  (10^-3.3±0.2^) | 8.1 ± 0.7 | n/a |
| EF123  (to misfolded) | 141 ± 8 | 4.4 x10^7^  (10^7.6±1.1^) | 1.4x10^-5^ | 2.3 x10^-8^  (10^-7.6±1.1^) | 16.6 ± 1.3 | 3.8x10^-5^ |
|  |  |  |  |  |  |  |
|  |  | k­_0, unfolding_ (s^-1^) | *p* value compared to RNC177 | τ_0, fold_ (s) | ∆x^†^_unfolding_ (nm) | *p* value compared to RNC177 |
| RNC177 |  | 0.5  (10^-0.3±0.1^) | n/a | 1.9  (10^0.3±0.1^) | 3.7 ± 0.4 | n/a |
| EF123  (to misfolded) |  | 5.9 x 10^-3^ (10^-2.2±0.9^) | 9.1x10^-4^ | 170  (10^2.2±0.9^) | 4.4 ± 1.6 | n.s. (0.22) |

Supplementary Table 3: Concentrations in *in vitro* translation mix for real-time elongation in tweezers

|  | Concentration |
| --- | --- |
| HEPES pH 7.5 | 20 mM |
| KCl | 95 mM |
| MgCl_2_ | 7.4 mM (5 mM “free”) |
| NH_4_Cl | 5 mM |
| CaCl_2_ | 0.5 mM |
| Spermidine | 1 mM |
| Putrescine | 8 mM |
| NaN_3_ | 10 mM |
| DTT | 1 mM |
| ATP | 1 mM |
| GTP | 1.4 mM |
| Creatine phosphate | 12 mM |
| S100 charged tRNA | 30 µM |
| Uncharged tRNA | 35 µM |
| EF-Ts | 1.5 µM |
| EF-Tu | 10 µM |
| EF-G | 10 µM |
| Creatine Kinase | 3.9 µg/mL |
| Myokinase | 3 µg/mL |
| NDPK | 0.8 µM |
| Pyrophosphatase | 1 µg/mL |
| RNase Out inhibitor | 0.15 U/uL |
| Alanyl tRNA synthetase | 0.4 µM |
| Arginyl tRNA synthetase | 0.5 µM |
| Asparaginyl tRNA synthetase | 0.2 µM |
| Aspartate tRNA synthetase | 0.3 µM |
| Cysteinyl tRNA synthetase | 0.5 µM |
| Glutaminyl tRNA synthetase | 0.05 µM |
| Glutamyl tRNA synthetase | 0.5 µM |
| Glycyl tRNA synthetase | 1 µM |
| Histidyl tRNA synthetase | 0.2 µM |
| Isoleucyl tRNA synthetase | 0.2 µM |
| Leucyl tRNA synthetase | 0.5 µM |
| Lysyl tRNA synthetase | 0.11 µM |
| Methionyl tRNA synthetase | 0.05 µM |
| Phenylalanyl tRNA synthetase | 0.8 µM |
| Prolyl tRNA synthetase | 0.6 µM |
| Seryl tRNA synthetase | 0.075 µM |
| Threonyl tRNA synthetase | 0.75 µM |
| Tryptophanyl tRNA synthetase | 1 µM |
| Tyrosyl tRNA synthetase | 0.2 µM |
| Valyl tRNA synthetase | 0.1 µM |

Supplementary Table 4: Sequences of primers used in this study

| **Name** | **Sequence (5’–3’)** |
| --- | --- |
| CaELy_fw | ATGCGGCCGCTATGACTACGGCCATCGCCAGC |
| CaELy_rv | ATGCTAGCGCCGAGCAGCTCGACGTC |
| Ef1_D29Q_rv | AGAGCACCGTTGCCGTCG |
| EF1_D29Q_fw | GGAGCGGGCGcagTTCGAGAAGG |
| CaE_Ndom_NheI_rv | ATGCTAGCGAAGATGAGGTTCTCGGTG |
| CaE_Cdom_NotI_fw | ATGCGGCCGCTGAGCAGGGCGAGGCCAGCTT |
| EF3_E124Q_r | ATCTGGCCGTCGGCGTTC |
| EF3_E124Q_f | CAACGCCGACcagTTCGCGGCGT |
| CaE123_NheI_rv | ATGCTAGCCATGCCCAGTGCGGTCAGCCAC |
| EF4_E158Q_r | AGCTCGCCGTTGCCGTTG |
| EF4_E158Q_f | GTCGCTCGACCAGCTGCTCACCG |
| RNC135_EF2_rv | GCTCATGCCCAGTGCGGTCA |
| RNC146_library1_1_rv | CACCTGGTTGAAGGCCTCC |
| RNC156_library1_2_rv | GTCGAGCGACAGCTCGCC |
| RNC167_library_3_rv | GTGGAAGTCACGCACGGCG |
| RNC177_EF3_rv_stop | GCCGAGCAGCTCGACGTC |
| RNC177+45_EF4_rv | AGCACGGATGCCTTGCGCCT |
| Fw_T7 | GTCGGCGATATAGGCGCCAG |
| Avi_SG+10_rv | gctgccggaacctgatccactgccagatccGGATCCACCGCCACCGGAGCC |
| CaE+10SG+3Val_fwd | ggtagcggcagcggtagcggtagcggcagcGTTGTAGTCATGACTACGGCCATCGCCAGC |
| internal dig oligo | (Dig)CGGGAGTGATTTCCGTCT(T-Dig)ACGGT |
| 1900 biolambda TI | (biotin) GAGTTTCCTGCTCCGTCTGA |
| 2kbH_BsaI-Dig_fw3 | atacggtgcGGTCTCaGTCGGAGTTTCCTGCTCCGTCTGA |
